# Effect of malnutrition at admission on length of hospital stay among adult surgical patients in Wolaita Sodo University Comprehensive Specialized Hospital, South Ethiopia: prospective cohort study

**DOI:** 10.3389/fnut.2024.1451463

**Published:** 2024-10-30

**Authors:** Zewdu Gebregziabher, Debritu Nane, Samson Kastro Dake, Yoseph Halala Handiso

**Affiliations:** Department of Reproductive Health and Nutrition, School of Public Health, College of Health Science and Medicine, Wolaita Sodo University, Sodo, Ethiopia

**Keywords:** effects of malnutrition, length of hospital stay, surgical patients, adults, Ethiopia

## Abstract

**Background:**

Malnutrition in hospitalized patients remains a major public health problem in both developed and developing countries. Even though malnourished patients are more prone to stay longer in hospital, there is limited data regarding the magnitude of malnutrition and its effect on length of stay among surgical in patients in Ethiopia while nutritional assessment is also often a neglected component of the health service practice.

**Objective:**

This study aims to assess the prevalence of malnutrition at admission and its effect on the length of hospital stay among adult surgical patients in Wolaita Sodo University Comprehensive Specialized Hospital, South Ethiopia, 2022.

**Methods:**

A facility-based prospective cohort study was conducted among 398 admitted surgical adult patients. Participants in the study were chosen using a convenient sampling technique. Subjective global assessment was used to determine the nutritional status of patients with a minimum stay of 24 h within 48 h after admission (SGA). Data were collected by open data kit (ODK) version 2022.3.3 software while Stata version 14.1 software was employed for statistical analysis. Cox regression model was used to determine the effect of malnutrition on the length of hospital stay (LOS) after adjusted for several potential confounders taken at admission. Adjusted hazard ratio (HR) with 95% confidence interval was used to show the effect of malnutrition.

**Results:**

The prevalence of hospital malnutrition at admission was 64.32% (95% CI: 59–69%) according to subjective global assessment (SGA) classification. Adult surgical patients who were malnourished at admission had higher median LOS (12 days: 95% CI: 11–13) as compared to well-nourished patients (8 days: 95% CI: 8–9), which means adult surgical patients who were malnourished at admission were at a higher risk of reduced chance of discharge with improvement (prolonged LOS) (AHR: 0.37, 95% CI: 0.29–0.47) as compared to well-nourished patients. The presence of comorbidity (AHR: 0.68, 95% CI: 0.50–90), poly medication (AHR: 0.69, 95% CI: 0.55–0.86), and history of admission (AHR: 0.70, 95% CI: 0.55–0.87) within the previous 5 years were found to be the significant covariates of LOS.

**Conclusion:**

The magnitude of hospital malnutrition at admission was found to be high. Malnourished patients at admission had a higher risk of prolonged length of hospital stay as compared to well-nourished patients. The presence of comorbidity, poly medication, and history of admission were found to be the significant covariates of LOS. All stakeholders should pay attention to reducing the magnitude of malnutrition and its covariates to improve the burden of LOS.

## 1 Background

The condition of imbalance in nutrition is called malnutrition.

Malnutrition is a significant global health issue, affecting populations worldwide. Malnutrition is a common problem among hospital patients worldwide, with estimates suggesting that up to 50% of hospitalized patients may be malnourished. The prevalence of malnutrition can vary depending on the patient population, hospital setting, and the specific criteria used to define malnutrition. Developed countries typically report malnutrition prevalence ranging from 20 to 50%, while developing countries often have higher rates, sometimes exceeding 50%.

Malnutrition is a significant public health concern in Ethiopia, affecting both the general population and hospital patients.

Studies have reported varying prevalence rates of malnutrition among hospitalized patients in Ethiopia, with estimates ranging from 20 to 57% (1). A deficiency, excess, or imbalance of a wide range of nutrients results in measurable adverse effects on body composition, function, and clinical outcome. Overnutrition and undernutrition can be the two reasons causing malnutrition (1, 2). Several factors may contribute to malnutrition such as underlying illness, socio-economic status, little knowledge of patients, certain diagnostic or therapeutic procedures, lack of standardized nutrition care, lack of monitoring of nutritional status, and other factors that impact food intake (3, 4). Although malnourished individuals can be under- or overnourished, in this study the term malnutrition refers to undernutrition.

Malnutrition in hospitalized patients still remains a major public health problem in both developed and developing countries (5, 6). Malnutrition is of special importance for the surgical patient due to its influence on postoperative patient outcomes (2). It is associated with various negative clinical outcomes including mortality, prolonged length of stay, readmission, delayed wound healing, impaired functional status, and other hospital-acquired conditions like surgical site infection (7, 8).

Malnutrition may affect all three phases: inflammation, proliferative, and remodeling of the wound-healing process due to vitamin and mineral deficiencies. Moreover, these unfavorable consequences cause a longer hospital stay and a delayed recovery, which raises the financial burden on patients and the healthcare system (9, 10). One of the most important performance metrics for hospital administration and the effectiveness of the healthcare system is the length of stay (11, 12). Additionally, it serves as an indirect indicator of malnourishment (11). Because it is linked to healthcare costs and may indicate a better clinical outcome and improved quality of life, it is frequently used to assess the efficacy of hospital care (10–12).

Different strategies have been devised to prevent malnutrition during a patient’s hospital stay. Some of those strategies include provision of food for hospitalized patients, nutritional screening, and assessment, which is usually done within the first 24 h of admission and nutritional interventions like enteral and parenteral nutrition (13). However, screening on admission is only the first step of the nutrition care process. Furthermore, routine screening with timely follow-up, employment of registered dietitians, appropriate nutritional intervention, and provision of more supportive mealtime are needed to prevent malnutrition during hospital stay (14).

Currently, more than 20 different tools are available to assess the risk of and diagnose malnutrition (15, 16). But the each tool has a different set of criteria and their accuracy of diagnosis and practicality in clinical settings vary (15). Some of the nutritional screening and assessment tools include malnutrition universal screening tool (MUST), mini nutrition assessment (MNA), Nutrition Risk Screening 2002 (NRS2002), and subjective global assessment (SGA) (14, 17). Among the nutritional screening tools, the most popular and well-known technique was found to be the SGA and it was designed to evaluate the nutrition risk of surgical patients who have complications arising due to infections (17, 18). SGA has been mainly used due to its simplicity, high sensitivity, non-invasiveness, low-cost, high-speed completion, and feasibility. It also has the ability to identify patients at a high nutritional risk in various clinical locations (2, 19).

Studies reported that up to 40% of patients are malnourished at the time of their admission and the majority of them continued to be nutritionally depleted throughout their hospital course (20). An observational study done in Iran among 353 patients undergoing an emergency surgery stated that 28% of the patients were at risk for malnutrition (21). Malnourished patients have longer hospital stay due to increased morbidity (22). According to a study conducted in Tikur Anbessa, 417 hospitalized patients who were malnourished spent considerably more time in the hospital (17.2 ± 6.8 days) than did well-nourished patients (8.3 ± 4.9 days) throughout the course of a 30-day observation period (4).

Even though malnutrition studies have been performed in developed nations, there is limited data regarding the prevalence of malnutrition in hospitalized patients. No such study has been conducted in southern Ethiopia to the best of our knowledge (20). Additionally, clinical nutrition and nutritional assessment are often the neglected components of the health service practice. Thus, the aim of this study is to determine the magnitude of malnutrition among hospitalized surgical patients on admission and its effect on their length of stay (20, 23).

### 1.1 Significance of the study

Studies have shown that early and adequate nutritional assessment and supplements significantly lower the length of stay and associated cost. Therefore, this study aims to assess the nutritional status and its effect on the length of hospital stay among the admitted adult surgical patients. Assessing the effect of malnutrition in hospitalized patients is assumes importance for ensuring wellbeing of patients and minimizing cost.

The findings will also provide information related to the necessity of addressing nutrition-related issues and drafting policies and strategies for integrating the nutritional aspect into the management of hospitalized patients. Healthcare professionals, the surgery department, hospital directors and board members, the regional health bureau, policymakers, and researchers will benefit from the information presented from this study. Healthcare providers will consider the importance of adding nutrition as one component of patient assessment and management after reviewing the results of this study.

Research Questions

1. What is the prevalence of malnutrition at admission among adult surgical patients?
2. Does malnutrition at admission have an effect on the length of hospital stay among adult surgical patients?
3. What are the factors that influence the length of hospital stay other than malnutrition?

## 2 Materials and methods

### 2.1 Study area and period

The study was conducted at the Wolaita Sodo University Comprehensive Specialized Hospital (WSUCSH) from June to July 2022, which is found in Wolaita Sodo town, Southern Ethiopia, about 329 km from Addis Ababa. WSUCSH is the only comprehensive specialized hospital in the Wolaita Zone and provides referral and non-referral services to about 15 million people. There are a total of 18 surgeons. An average of six surgeries are performed per day including major and minor surgeries and there are four surgery tables (two tables for minor surgery and two tables for major surgery).

The inpatient services include internal medicine, pediatrics, gyn/obs, psychiatry, surgery, and orthopedics. In surgery, there are also subunits like general surgery, laparoscopy, neurosurgery, maxillofacial, and uro-surgery. The study was conducted in the surgery department, including all the subunits.

### 2.2 Study design

An institution-based prospective cohort study design was conducted for 2 months from June to July 2022.

### 2.3 Population

#### 2.3.1 Source population

All adult surgical patients admitted to the surgical ward in WSUCSH.

#### 2.3.2 Study population

The selected adult surgical patients admitted to the surgical ward of WSUCSH from June to July 2022.

#### 2.3.3 Study unit

A single individual (an adult surgical patient) who was selected to participate in the study was considered as a study unit.

### 2.4 Inclusion and exclusion criteria

#### 2.4.1 Inclusion criteria

Adult surgical patients (>18 years of age) admitted to the surgical ward with a minimum length of stay of 24 h in the ward in WSUCSH from June to July 2022 were included in the study.

#### 2.4.2 Exclusion criteria

Adult surgical hospitalized patient (>18 years) who cannot communicate due to being in severe pain, unconscious, or clinically unstable, emergency surgery, and adult surgical patients who cannot stand were excluded from the study.

#### 2.4.3 Sample size determination

The sample size was calculated for both the first and second objectives. Finally, the maximum sample size was taken as the sample size of the study.

##### 2.4.3.1 Sample size for the first objective

To assess the magnitude of hospital malnutrition among adult hospitalized patients, the sample size was calculated using the single-population proportion formula as follows:


$$\frac{N=Z\;{\left(\alpha /2\right)}^{2}p\;\left(1-p\right)}{d^{2}}$$

$$\frac{{\left(1.96\right)}^{2}0.621\;\left(1-0.621\right)=362}{0.05^{2}}$$

After adding a non-response rate of 10%, the final sample size was 398.

Where, Z (*α* /2) is standard normal distribution with a confidence interval (CI) of 95% and α = 0.05, P is the expected proportion of hospital malnutrition, which is 62.1% (from a study done in Tikur Anbessa) (4) and d is the absolute error or precision = 0.05.

##### 2.4.3.2 Sample size for the second objective

To determine the effect of malnutrition on the hospital length of stay among adult surgical patients, the sample size was calculated using double-population proportion formula as follows:


$$\frac{N=[Z\alpha /2\surd (1\pm 1/r\pm Z\beta \surd p1\;\left(1-p1\right)\pm p2\;(1-p2/r]2}{{\left(P1-p2\right)}^{2}}$$

Where Zα/2 is the probability of type 1 error (=1.96), *β* is the probability of rejecting a true difference (=20%), and r is the proportion of n1 to n2 (=1).

Since the sample size calculated for the first objective (398) was greater than the sample size calculated for the second objective (267), the maximum sample size calculated for the first objective (398) was taken as the final sample size of the study to increase the statistical power and representativeness of the study (Table 1).

**Table 1:** Sample size determination for the effect of malnutrition at admission on length of hospital stay among adult surgical patients in WSUCSH, South Ethiopia, 2022.

P1 (Proportion of prolonged length of hospital stay)	P2 (Proportion of prolonged length of stay among well-nourished patients)	P (Pooled proportion)	R	Α	Β	Non response rate	sample size
37.2%	14.1%	20%	1	0.05	20%	10%	267

### 2.5 Sampling procedures

In WSUCSH, there are approximately 46 beds for surgical patients and it receives approximately 355 surgical patients within one month. For this study, 398 patients are needed. Non-probability sampling method (convenient method) was used to select the study participants. As a result, all consecutively admitted surgical patients to the surgery ward with a minimum length of stay of 24 h and who fulfill the inclusion criteria were recruited for the study until the required sample size was reached during the data collection period.

### 2.6 Study variables

#### 2.6.1 Dependent variables

- Lengths of hospital stay

#### 2.6.2 Independent (exposure) variable

- Nutritional status (SGA)

#### 2.6.3 Covariates

##### 2.6.3.1 Socio- demographic

- Age sex, occupation, marital status, and residence

##### 2.6.3.2 Disease and surgical-related

- Primary diagnosis on admission
- Presence of comorbidity
- Diagnosis of cancer
- Previous history of surgery

##### 2.6.3.3 Baseline clinical-related

- Number of drugs at admission
- Previous admission

### 2.7 Operational definitions

- **Event**: Discharge of patients with improvement (recovery) (4).
- **Censored**: Patients who left the hospital against medical advice, dead, referred to other institution without clinical improvement, or stayed in the hospital for more than 60 days (4).
- **Lengths of hospital stay**: The time difference in days between date of admission and date of discharge. It was calculated by subtracting the date of admission from date of discharge and documenting the total number of days that the patient stayed in the hospital. Patients who left the hospital against medical advice, dead, referred to other institution without clinical improvement, or stayed in the hospital more than 60 days were considered as censored observations (20).
- **Hospital malnutrition**: Malnutrition identified during hospital admission (7).
- **Surgical inpatients**: Patients who are admitted to surgical ward for surgical intervention (24).
- **Comorbidity:** Additional medical illness other than the primary surgical diagnosis (22).

Subjective global assessment (SGA) consists of medical history and physical examination. In medical history, there are five components to be asked. These include weight change, dietary intake, gastrointestinal symptoms, metabolic demand, and functional capacity. The physical examination component of SGA includes subcutaneous fat, muscle tone and bulk, edema/fluid overload, and noted as either normal (0), mild (1 +), moderate (2+), or severe (3+). Based on the scores given for each medical history and physical examination components, SGA was ranked as SGA-A (well-nourished), SGA-B (moderately or suspected malnourished), or SCA C (severely malnourished).

The seven-point SGA provided later was used to classify malnutrition as well nourished (6–7), mildly/moderately malnourished (3–5), and severely malnourished (1–2).

Even though non-probability sampling method (convenient method) was used to select the study participants, all consecutively admitted patients were taken for the study. This will reduce researcher bias and it can also improve the reliability and validity of individuals.

**SGA history components**

- The following formula was used to compute weight loss.
  (Normal weight (kg) – current weight) / usual weight = 100
- Using an electronic portable scale, weight was recorded to the nearest 0.1 kg while wearing little clothing and no shoes (Seca, Hamburg, Germany). Prior to each weigh-in, the scale was tested for a zero reading to guarantee measurement accuracy. The patient’s pre-illness weight, as reported by him, was utilized to calculate the percentage of weight loss (PWL). PWL is determined by dividing the typical weight by the horsepower and subtracting the present weight from the usual body weight in kilograms.
- Dietary intake was determined if there is decreased intake over a specific period of time:
- (Significant if <50% plate)
- Gastrointestinal symptoms include nausea, vomiting, dysphagia, and diarrhea
- Metabolic demand: Patients with inflammatory diseases (e.g., infections) are likely to become malnourished sooner due to muscle breakdown
- Functional capacity: Patients who are malnourished are frequently less mobile (ambulatory, bedridden)

**Components of SGA physical examination**

- Subcutaneous fat: The following area were assessed:
- Triceps
- Muscle tone and bulk: The following muscles were assessed:
- Temporalis muscle, interossei and shoulder area
- Edema/fluid overload

Ankle edema, sacral edema, and ascites.

**Subjective global assessment nutritional status classification**

- **Well-nourished**: No decrease in food/nutrient intake; <5% weight loss; no/minimal symptoms affecting food intake; no deficit in function; no deficit in fat or muscle mass
- **Mildly/moderately malnourished**: Definite decrease in food/nutrient intake; 5–10% weight loss without stabilization or gain; mild/some symptoms affecting food intake; moderate functional deficit or recent deterioration; mild/moderate loss of fat and/or muscle mass
- **Severely malnourished**: Severe deficit in food/nutrient intake; >10% weight loss, which is ongoing; significant symptoms affecting food/ nutrient intake; severe functional deficit (17)

### 2.8 Data collection tool and procedures

Data were collected electronically using smartphones through ODK version 2022.3.3 software by pretesting and the questionnaire was developed from different studies (17, 25).

The questionnaire was prepared in English language and then it was translated to Amharic language. Finally, it was translated back to English to maintain conceptual consistency by a language expert and health professional. The nutritional status of the patients was assessed within 48 h of admission through the use of nutritional assessment tool, SGA. SGA classifies the patients’ nutritional status in three degrees: well nourished, mild to moderate (or suspected of being undernourished), or severe under nutrition.

The data were collected at the surgical ward for 2 months by health professionals who had experience in data collection and ODK use (three BSc nurses who can speak Amharic and Wolaitigna) under the supervision of the principal investigator.

**Anthropometric measurements:** Weight was determined with a calibrated floor SECA® scale, with the patients shoeless and only wearing light cloth, to the nearest of 0.1 kg.

### 2.9 Data quality assurance

The data collection tool was first prepared with pre-coded skip patterns (relevancy), must-fill restrictions (requirement), pre-coded ranges (constraint), and pre-coded types of data (type) in a Microsoft Excel sheet, which were an important initial step to maintain the quality of data. Then the prepared tool was validated through the uploading and deploying strict sequential tool validating procedures of the KoBo Collect humanitarian response website (server) and it was approved for the validation procedures and requirements of the server. In addition to this, a pretest was conducted by the principal investigator before the actual data collection in Sodo Christian General Hospital on 5% of the sample size (20 patients) and the data collected from the pretest were not included in the study.

Moreover, data collectors and supervisors took theoretical and practical training for 2 days to have a common understanding about the aim of study, techniques of data collection, procedures to be followed, contents of the tool, how to use open data kit (ODK) software, and the in which weight and height measurements are taken.

Completeness, consistency, and accuracy of the data were checked by data collectors, supervisors, and the principal investigator every day. After checking for completeness, consistency, and accuracy, data collectors submitted every filled tool automatically to the Kobo toolbox aggregator (server) and the data were stored in the cloud storage server of the KoBo Collect humanitarian response website, which provided free data storage spaces for the principal investigator.

The pre-coded skip patterns, data types, ranges, and restrictions in the ODK Collect greatly helped to maintain quality of data and reduce errors throughout the data collection period. Similarly, ODK Collect helped to control the daily data collection process remotely. Incorrectly filled data were identified every day and correction was done by respective data collectors. Each medical record was labeled with a unique identification code, which was used during data management. The whole data collection process was also closely monitored by the supervisors.

**Figure 1:**
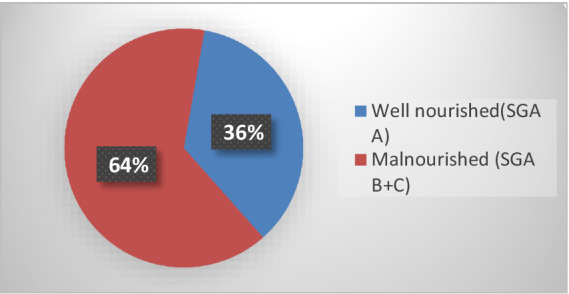
Magnitude of malnutrition by subjective global assessment (SGA) among adult surgical patients at admission in WSUCSH, southern Ethiopia.

### 2.10 Data management and statistical analysis

Data was collected by ODK Collect version 2022.3.1 software and it was checked, coded, cleaned, and refined. The data collected via ODK was exported and downloaded in XLS format. Then downloaded data were imported to Stata version 14.10 for statistical analysis. Data cleaning, labeling, and recoding were done. Descriptive statistics were used to summarize data in the form of tables, graphs, and numerical measures. Kaplan Meier analysis was used to describe the survival of patients from being discharged among the different categories of nutritional status using survival curves and log-rank pairwise comparison. Cox regression model was used to investigate the effect of malnutrition on the length of hospital stay after adjusted for several potential nutritional and clinical confounders recorded at admission. Proportional hazard assumption was also checked by using log–log survival curves and correlation test. Categorical variables for multivariable analysis were selected using *p* value for pooled over strata log-rank test. Those categorical variables with a *p* value of 0.25 or less were selected for multivariable analysis. As for continuous variables, p value of the Wald test for coefficient 0.25 or less during bivariate analysis was the criteria. Before fitting the Cox regression model, the absence of multicollinearity was ensured using variance inflation factor (VIF). All predictors had VIF less than five. The mean value of VIF was 1. 04, which is less than 5.

### 2.11 Ethical considerations

The Ethical Review Committee of Wolaita Sodo University’s Department of Public Health granted ethical approval for the study, and prior to data collection, a letter of permission was acquired from the hospital’s medical director and surgical ward matron.

Study participants were interviewed to get an informed written consent that assures the willingness of each participant to participate in the study. The data collectors read out and explained the information to non-literate participants and obtained written consent from their witnesses on their behalf. To ensure privacy, any study subject was left free to withdraw from the study at any time they want and confidentiality was ensured by not mentioning names of the study participants and through the use of strict coding measures.

## 3 Results

### 3.1 Socio-demographic characteristics

A total of 398 hospitalized surgical patients were included in the study. The majority (224 (56.28%)) of the study participants were between 19 and 40 years age group while only 31 (7.79%) participants were older than 64 years of age. The median age of the participants was 36 years (**IQR: 26–56** years). Similarly, about 243 (61.06%) study participants were men and 240 (60.3%) of them were rural dwellers. And 249 (62.56%) participants were married while 3.02% of them had divorced. Moreover, about 101 (25.38%) participants had primary level of educational status while 17.84% have had college education and above (Table 2).

**Table 2:** Socio-demographic characteristics of the adult surgical patients at admission in WSUCSH, south Ethiopia, 2022 (*N* = 398).

Variable	Category	Frequency	Percent
Gender	Men	243	61.06
	Women	155	38.94
Age (in complete years)	19–40	224	56.03
	41–64	144	36.18
	> = 65	31	7.79
Marital status	Single	123	30.9
	Married	249	62.56
	Divorced	12	3.02
	Widowed	14	3.52
Occupational status	Farmer	114	28.64
	Housewife	92	23.12
	Government employee	51	12.81
	Merchant	32	8.04
	Student	97	24.37
	Others	12	3.02
Educational status	No formal education	110	27.64
	Primary (1–8)	101	25.38
	Secondary (9–12)	116	29.15
	College and above	71	17.84
Residence	Rural	240	60.3
	Urban	158	39.7

### 3.2 Clinical- and surgical-related characteristics at admission

The majority (150 (37.69%)) of the patients enrolled in this study were hospitalized with primary diagnosis of gastrointestinal causes while a few of them [14 (3.52%)] were admitted for neurological causes. As many as 65 (16.33%) participants had secondary diagnosis (co-morbidity) in addition to the primary diagnosis, of which majority [27 (41.5%)] of them were hypertensive patients followed by 12 (18.46%) patients with diabetes. Moreover, 123 (30.90%), 47 (11.81%), and 21 (5.28%) participants have history of admission, history of surgery, and diagnosis of cancer, respectively (Table 3).

**Table 3:** Clinical and surgical related characteristics at admission of the adult surgical patients in WSUCSH, south Ethiopia, 2022.

Variable	Category	Frequency	Percent
Primary diagnosis	GI	150	37.69
	GU	60	15.08
	Hematology	19	4.77
	Endocrine	35	8.79
	MSK	32	8.04
	Neurologic	14	3.52
	Respiratory	43	10.80
	Trauma	45	11.31
Comorbidity (N = 65)	Yes	65	16.33
	No	333	83.67
History of admission	Yes	123	30.90
	No	275	69.10
History of surgery	Yes	47	11.81
	No	351	88.19
Diagnosis of cancer	Yes	21	5.28
	No	377	94.72
Number of drugs at admission	<5 drugs	371	93.22
	> = 5drugs	27	6.78

### 3.3 Magnitude of hospital malnutrition by subjective global assessment (SGA)

Out of a total of 398 adult hospitalized patients who participated in the study, 256 (64.32%) of them were malnourished while 142 (35.68%) were well nourished according to the SGA assessment (Figure 1).

### 3.4 Length of hospital stay

**Figure 2:**
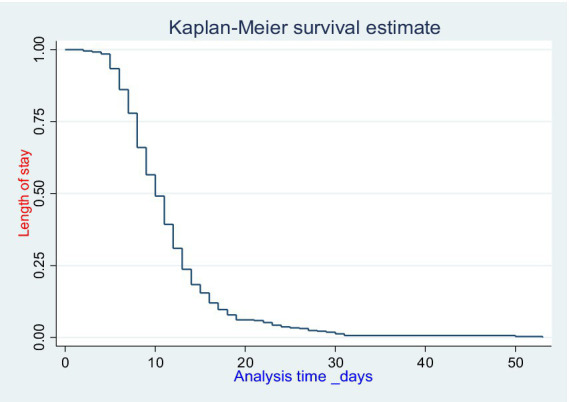
Overall Kaplan–Meier estimates of length of hospital stay among adult surgical patients in WSUCSH, south Ethiopia.

The total time observed in the study was found to be 4,482 days with a median follow-up time of 10 days (IQR: 8–13 days). Similarly, during the study period, a total of 369 [92.71% (95% CI: 89.69–94.89%)] participants were discharged with improvement and 29 [7.29% (5.10–10.30%)] of the participants were censored (Figure 2).

### 3.5 Comparison of survival experience within (intra) different categorical predictors

Kaplan–Meier survival curve was used to estimate the survival probability of category of each predictor while log rank test was used to compare survival experiences within the categorical variables along with *p*-values. Based on the Kaplan–Meier and log rank test, significant proof of difference in survival experience had denoted the categories of nutritional status, comorbidity, Hx of admission, and number of drugs (Table 4).

**Table 4:** Comparisons of survival experiences within different categorical variables by using log rank test for the assessment of the effect of malnutrition on length of hospital stay among adult surgical patients in WSUCSH, south Ethiopia, 2022 (*n* = 398).

Variables	Log-rank (*x*^2^)	*p*-value	Variables	Log –rank (*x*^2^)	*p*-value
Nutritional status (SGA)	138.83	<0.001	Hx of admission	6.98	0.0082
Age	4.81	0.0902	Number of Drugs	13.17	< 0.001
sex	1.01	0.3139	Primary Dx	20.30	0.0050
Residence	2.41	0.1208	Co-morbidity	13.40	0.0199
Marital status	3.10	0.0678	Dx of cancer	1.42	0.2333
Educational status	6.66	0.0836	Hx of surgery	2.47	0.1161
Occupation	2.29	0.110			

**Figure 3:**
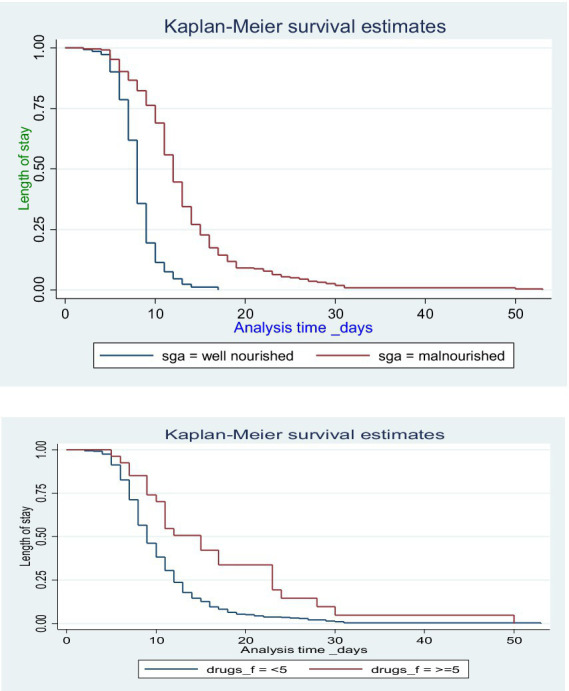
Kaplan–Meier survival estimates of the length of hospital stay by nutritional status and number of medications among hospitalized adult surgical patients.

The length of hospital stay was found to be higher within malnourished patients, history of admission, who took more than five drugs, and within those who have comorbidity (Figure 3).

### 3.6 Cox proportional hazard assumption and model fitness

**Figure 4:**
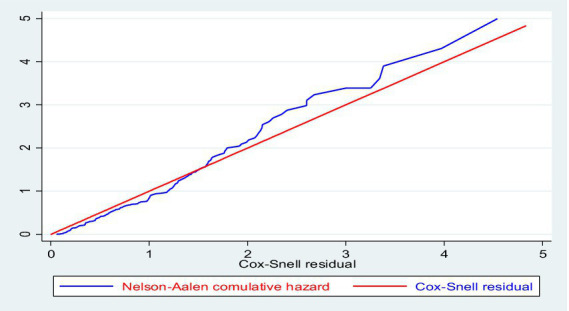
Nelson–Aalen cumulative hazard against Cox–Snell residuals of adult surgical patients.

The overall Schoenfeld global test of full model was checked for proportional hazard assumption of all variables in the final model and the null hypothesis was not violated (*x^2^* = 9.24, df = 20, *p* = 0.9800) (Table 5). The model fitness was checked by plotting Nelson–Aalen cumulative hazard with Cox–Snell residual and the Cox–Snell residual was almost in line with the Nelson–Aalen cumulative hazard. The residuals had standard censored exponential distribution with hazard ratio and the hazard function follows the 45° line very closely (Figure 4).

**Table 5:** The overall Schoenfeld global test of full model of proportional hazard assumption of all variables in the final model for the assessment of effect of malnutrition on the length of hospital stay among adult surgical patients.

Variables		Rho	Chi^2^	df	Prob > chi^2^
Nutritional status (by SGA)	Well nourished	Reference		1	
	Malnourished	0.00448	0.000	1	0.9490
Marital status	Single	(reference)		1	
	Married	0.00524	0.01	1	0.9358
	Widowed	0.04390	0.39	1	0.5315
	Divorced	−0.04975	0.51	1	0.4760
Occupation	Farmer	(reference)		1	
	Housewife	0.01093	0.02	1	0.8765
	Employee	−0.01829	0.08	1	0.7829
	Merchant	0.01241	0.03	1	0.8604
	Student	−0.01582	0.05	1	0.8153
	Others	0.02742	0.16	1	0.6849
Previous history of admission	No	(reference)		1	
	Yes	−0.05275	0.57	1	0.4498
comorbidity status	No	(reference)			
	Yes	−0.13369	3.47	1	0.0623
Number of medications	Less than five	(reference)		1	
	Greater than or equal to five	−0.05621	0.66	1	0.4172

### 3.7 Nutritional risk status and length of hospital stay

Sociodemographic, clinical, and surgical covariates were fitted to assess the effect of malnutrition (SGA B + C) at admission on the length of hospital stay. On bi-variable Cox regression model age, presence of comorbidity, number of medications, and history of admission with a *p*-value of ≤0.25 were selected to multivariable Cox regression. Whereas sex, residence, educational status, occupation, marital status, diagnosis of cancer, and history of surgery with p-value >0.25 in bi-variable Cox regression were not eligible for the multivariable Cox regression model. Moreover, malnourished patients had a longer hospital stay as compared to well-nourished patients (*p* < 0.001).

The final multivariable model, which was controlled for age, number of medications, presence of comorbidity, and history of admission showed that malnutrition (SGA B + C) at admission was independently associated with prolonged length of hospital stay in which patients who were malnourished at admission had about 63% reduced chance of discharge (chance of staying in hospital) [(AHR, 0.37; 95% CI (0.29–0.47)] as compared to well-nourished patients at admission.

Patients who had additional comorbidity other than the primary diagnosis had about 32% higher risk of staying in the hospital longer (less chance of discharge) (AHR: 0.68, 95% CI: 0.50–0.90) as compared to those with no additional comorbidity. Similarly, patients who took more than or equal to five medications had 31% reduced chances for discharge (delayed stay) (AHR: 0.69, 95% CI: 0.55–0.86) as compared to those who were taken less than five medications. Moreover, adult surgical patients who had history of previous admission within the past 5 years had about 30% higher risk of longer hospital stay (reduced chance of discharge) (AHR: 0.70, 95% CI: 0.55–0.87) as compared to those who had no history of admission within the past 5 years (Table 6).

**Table 6:** Multivariable Cox regression model for the effect of malnutrition at admission on the length of hospital stay among adult surgical patients.

Covariates	Median length of stay (Days)	CHR (95% CI)	*p*-value	AHR (95% CI)	*p*-value
Nutritional status at admission					
Well-nourished (SGA A)	8 (8–9)	1.00 (reference)		1.00 (reference)	
Malnourished (SGA B + C)	12 (11–13)	0.38 (0.31–0.48)	<0.001	0.37 (0.29–0.47)	<0.001
Age					
19–40 years	10 (9–11)	1.00 (reference)			
41–64 years	11 (10–11)	0.84 (0.67–1.04)	0.124	0.88 (0.70–1.10)	0.285
≥ 65 years	13 (10–14)	0.73 (0.49–1.06)	0.106	0.74 (0.50–1.09)	0.130
Presence of comorbidity					
No	10 (10–11)	1.00 (reference)		1.00 (reference)	
Yes	13 (12–15)	0.61 (0.462–0.813)	0.001	0.68 (0.50–0.90)	0.009
Number of medications					
< 5	10 (9–10)	1.00 (reference)		1.00 (reference)	
≥ 5	11 (11–12)	0.56 (0.46–0.70)	<0.001	0.69 (0.55–0.86)	0.001
History of admission					
No	10 (9–11)	1.00 (reference)		1.00 (reference)	
Yes	12 (11–12)	0.72 (0.57–0.89)	0.003	0.70 (0.55–0.87)	0.002

## 4 Discussion

Malnutrition can contribute to longer hospital stays for patients through several interrelated mechanisms. Firstly, malnutrition can impair the immune system, making patients more susceptible to infections and complications, which can prolong the recovery time. Malnutrition can also impair wound healing and tissue repair, leading to complications and delayed recovery. In addition to these issues, malnourished patients may experience muscle weakness and decreased functional status, hindering their ability to participate in rehabilitation and delaying their progress. Malnutrition can also slow down the body’s response to medical interventions, decreasing treatment efficacy, and prolonging the hospital stay. Finally, malnourished patients have a higher risk of hospital readmission, further contributing to longer cumulative hospital stays.

The aim of this study was to determine the effect of malnutrition at admission on the length of hospital stay among adult surgical patients at WSUCSH, southern Ethiopia, 2022.

This study found that the overall prevalence of hospital malnutrition at admission by SGA classification was 64.32% (95% CI: 59–69). This finding was consistent with studies conducted in Tikur Anbessa specialized hospital, Adiss Ababa (62%) (4) and Vietnam (64.7%) (10).

However, the finding of this study was found to be higher as compared to studies conducted in Amhara National Regional State Referral Hospitals (55.6) (26), Ankara (Turkey) (46.5%) (6), Korea (49.5%) (1), and the Netherlands (42%) (16). This difference might be probably due to variation in the method of assessment tool used. Similarly, this difference might be due to the variation in the duration of follow-up period, differences in the socioeconomic status, and study design.

This study also revealed that adult surgical patients who were malnourished at admission had a higher risk of length of hospital stay (reduced chance of discharge) [AHR, 0.37; 95% CI (0.29–0.47)] as compared to those who were well nourished at admission. This finding was supported by studies conducted in northern Ethiopia (11, 27), Tikur Anbessa (Adiss Ababa) (4), Ghana (8), and Korea (1), which revealed that those who were malnourished at admission were significantly associated with a prolonged hospital length of stay.

The study also found that the presence of comorbidity was significantly associated with a prolonged length of hospital stay (AHR: 0.68, 95% CI: 0.50–0.90). This finding was supported by studies conducted in Tikur Anbessa, Adiss Ababa (4). This might be due to the fact that the presence of comorbidities impairs the immune function, which in turn results in a delayed discharge. This study also showed that patients who were taking poly medications (five or more medications) were at higher risk of prolonged length of hospital stay as compared to those who were taking less than five medications. This finding was in line with a study conducted in Addis Ababa, Ethiopia (4, 28, 29). This might be probably due to the reason that taking multiple medications is suspected to cause adverse reactions; for example, it can cause cognitive decline and inhibit metabolism, resulting in an increase in blood concentration leading to a prolonged length of hospital stay. In addition to this, those who took poly medication have multiple underlying diseases and to treat all of them, the therapy period may be prolonged. Moreover, patients who have a previous history of admission within the previous 5 years were at higher risk of prolonged length of hospital stay as compared to those who have no history of previous admission.

### 4.1 Strength

Since this study used SGA to assess malnutrition among the hospitalized surgical patients, it has the advantage of better validity. Similarly, since this study is a prospective cohort study, it has an advantage of showing temporal relationship and also shares other advantages of prospective studies.

### 4.2 Limitation

A socio-economic variable like level of income was not included in the study. Using non-probability sampling method is the limitation of this study as it is not representative and cannot be generalized. This study only accesses malnutrition at admission and it does not consider the effect of hospital-acquired malnutrition during hospital stay. Even though the power of SGA is valid and strong, the actual body weight is often a guess as patients do not weigh themselves regularly.

## 5 Conclusion and recommendation

### 5.1 Conclusion

The study revealed a notable prevalence of malnutrition upon hospital admission, indicating a significant occurrence within the patient population. Notably, malnourished individuals exhibited an extended length of hospital stay in comparison to their well-nourished counterparts. Moreover, factors such as the presence of comorbidities, the use of multiple medications (five or more medications), and a history of previous hospital admissions were identified as significantly linked to prolonged hospital stays among adult surgical patients at WSUCSH.

This research underscores the impact of malnutrition on hospitalization duration, highlighting the need for early identification and intervention strategies to address malnutrition in hospitalized patients effectively. The findings emphasize the interplay between malnutrition, comorbidities, medication burden, and past hospitalization history in influencing the length of hospital stay among adult surgical patients at WSUCSH, underscoring the importance of comprehensive nutritional assessment and management protocols in optimizing patient outcomes and healthcare resource utilization.

### 5.2 Recommendation

#### 5.2.1 Implementation of malnutrition screening protocols

Develop and implement hospital-wide malnutrition screening protocols to identify at-risk patients early in their hospital stay. Standardized screening tools such as the Malnutrition Screening Tool (MST) or the Malnutrition Universal Screening Tool (MUST) can be utilized.

#### 5.2.2 Integration of nutrition into hospital policies

Integrate nutrition assessment and management into hospital policies and protocols to ensure that all patients are routinely screened for malnutrition and receive appropriate nutrition care plans.

We recommend that patients at the time of hospital admission need to be routinely evaluated for malnutrition in hospitals and get adequate nutritional support based on their nutritional status. Nutrition must be integrated into the management of patients and nutritional therapy should start soon after identifying the patients who are at risk of malnutrition or malnourished.

#### 5.2.3 Nutrition assessment and diagnosis

Use validated tools such as the Subjective Global Assessment (SGA) or the Mini Nutritional Assessment (MNA) to assess the nutritional status of hospitalized patients accurately. Ensure that nutrition assessments are conducted promptly upon admission.

#### 5.2.4 Individualized nutrition care plans

Develop individualized nutrition care plans for patients identified as malnourished or at risk of malnutrition. Consider factors such as dietary preferences, comorbidities, and nutritional requirements when designing nutrition interventions.

Further interventional studies should be conducted in the future.

## Data Availability

The raw data supporting the conclusions of this article will be made available by the authors, without undue reservation.
